# Proteomic Analysis of the Mitochondrial Responses in P19 Embryonic Stem Cells Exposed to Florfenicol

**DOI:** 10.3390/toxics11120992

**Published:** 2023-12-06

**Authors:** Zhihua Dong, Xueke Hou, Xueying Wang, Zihui Shen, Huiqing Pang, Lingli Chen, Zhihong Yin, Fei Ren, Weiguo Li, Yaming Ge, Hongmei Ning, Dongfang Hu

**Affiliations:** 1 College of Animal Science and Technology, Henan Institute of Science and Technology, Xinxiang 453003, China; dzh@stu.hist.edu.cn (Z.D.); hxk15039417436@163.com (X.H.); wxy9910@163.com (X.W.); zihuishen2023@163.com (Z.S.); huiqingpang1027@163.com (H.P.); llchen2011@126.com (L.C.); yinhonghong1@163.com (Z.Y.); gghappy@126.com (F.R.); ndgeyaming@126.com (Y.G.); 2 Postdoctoral Research Station in Biological Sciences, Henan Normal University, Xinxiang 453003, China; liwg0618@htu.edu.cn; 3 Postdoctoral Research and Development Base, Henan Institute of Science and Technology, Xinxiang 453003, China

**Keywords:** florfenicol, proteomic analysis, mitochondrial dynamics, embryonic toxicity

## Abstract

Florfenicol (FLO) has been shown to elicit diverse toxic effects in plants, insects, and mammals. Previously, our investigations revealed that FLO induced abnormal cardiac development and early embryonic mortality in chicken embryos. However, the effect of FLO on mitochondrial responses in stem cells remains unclear. In this study, we show that FLO significantly diminishes proliferation viability and obstructs the directed differentiation of P19 stem cells (P19SCs) into cardiomyocytes. Proteomic analysis revealed 148 differentially expressed proteins in response to FLO. Functional analysis has pinpointed FLO interference with biological processes associated with oxidative phosphorylation within the mitochondria. In alignment with the results of proteomic analysis, we confirmed that FLO inhibits the expression of both nuclear DNA-encoded and mitochondrial DNA-encoded subunits of the electron transport chain. Subsequent experiments demonstrated that FLO disrupts mitochondrial dynamics and induces the mitochondrial unfolded protein response to maintain mitochondrial homeostasis. These findings collectively highlight the significance of mitochondrial dynamics and the mitochondrial unfolded protein response to mediate the decreased proliferation viability and directed differentiation potential in P19SCs treated with FLO. In conclusion, this study provides a comprehensive overview of mitochondrial responses to FLO-induced cytotoxicity and enhances our understandings of the molecular mechanisms underlying FLO-induced embryonic toxicity.

## 1. Introduction

Since the initial clinical use of sulfonamides and penicillins, a plethora of antibiotics have been developed and widely applied in both human and veterinary medicine, making substantial contributions to human well-being [1]. Moreover, antibiotics can promote the growth of edible animals and enhance feed efficiency [2]. Nonetheless, the extensive use of antibiotics has precipitated grave public health concerns. On the one hand, the indiscriminate use of antibiotics has engendered bacterial resistance, posing a significant threat to human health [3]. On the other hand, following absorption within the body, antibiotics are predominantly excreted via feces and urine, subsequently infiltrating the aquatic environment, thereby engendering profound ecological contamination [4,5].

Florfenicol (FLO), an innovative broad-spectrum antibacterial agent akin to chloramphenicol, has emerged as a pivotal therapeutic tool for the prevention and treatment of diverse animal maladies, exhibiting notable efficacy in combating bacterial infections in livestock and poultry caused by susceptible bacteria [6,7]. The antibacterial properties of FLO hinge on its ability to bind to ribosomes, thereby curbing peptidyl transferase activity and impeding microbial protein synthesis. Furthermore, it has been documented that FLO can also engage mitochondrial ribosomes, inhibiting mitochondrial protein synthesis [8]. FLO has been extensively deployed in the prevention and management of bacterial ailments, with some countries permitting its incorporation into livestock and poultry feed to augment feed conversion rates and growth. Additionally, FLO is capable of suppressing the production of various inflammatory factors and responses in the body [9,10]. Nonetheless, recent studies have increasingly unveiled the reproductive and embryonic toxicity of FLO [11]. Notably, it has been reported that administering FLO to hens significantly diminishes the hatching rate of eggs [12]. Our prior investigations corroborated that FLO leads to early-stage mortality in chicken embryos, manifesting conspicuous developmental retardation [13]. To date, the precise mechanisms governing FLO-induced embryonic developmental toxicity remain elusive.

The antibacterial effect of amphenicol drugs is rooted in their interference with the protein synthesis apparatus of bacteria, specifically ribosomes. Animal cells host two types of ribosomes: cytoplasmic and mitochondrial ribosomes. It is well documented that mitochondrial ribosomes bear greater structural and chemical similarities to bacterial ribosomes than their cytoplasmic counterparts, primarily due to the endosymbiotic origin of mitochondria from archaea [14]. Given the common lineage and structural resemblances between mitochondrial ribosomes and bacterial ribosomes, many antibacterial drugs targeting bacterial mitochondria inevitably hamper the translational function of eukaryotic mitochondrial ribosomes while exerting antibacterial effects, culminating in mitochondrial impairment [15]. An escalating body of research substantiates the pivotal role of mitochondria in the regulation of intricate biological processes (BP), including cell division, proliferation, apoptosis, embryonic development, pluripotency, and stem cell differentiation potential [16,17,18]. Consequently, mitochondrial damage plays a significant role in the toxicity and adverse effects of FLO. Drawing from previous reports and our research, it is plausible that FLO-induced embryo toxicity may be intricately linked to mitochondrial dysfunction.

P19 stem cells (P19SCs), characterized by pluripotency similar to embryonic stem cells, represent pioneering pluripotent embryonic stem cell lines frequently employed as an in vitro model system for studying early cellular development [19,20]. To unravel the molecular mechanisms underlying chemical-induced stem cell toxicity, iTRAQ-based proteomics analysis has emerged as an invaluable approach for dissecting differentially expressed proteins (DEPs) at the proteomic level. In this study, we explored the repercussions of FLO on mitochondrial proteomics and elucidated the associated functional alterations in P19SCs, a domain hitherto unknown. Consequently, we meticulously examined the effects of FLO on P19SC proliferation and differentiation, identified DEPs, performed bioinformatics analysis, and probed the relationship between mitonuclear protein equilibrium and mitochondrial stress in FLO-treated P19SCs. This investigation provides valuable insights into the judicious application of FLO, enriches toxicological knowledge, and establishes a foundation for further exploration of the mechanisms governing FLO-induced embryonic toxicity.

## 2. Results

### 2.1. FLO Inhibits P19SC Proliferation by Perturbing G1/S Phase Transition

**Figure 1:**
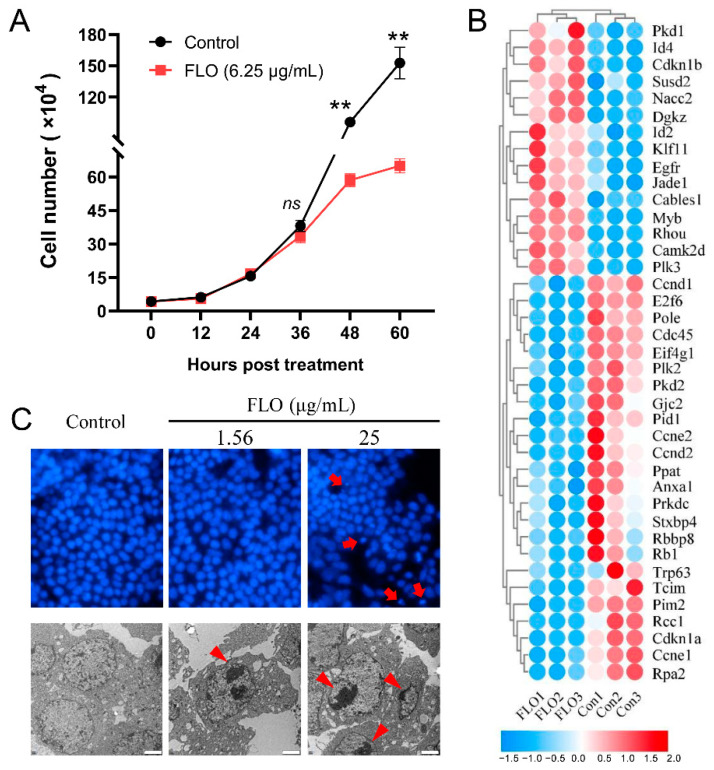
FLO inhibits P19SC proliferation by regulating G1/S phase transition. (**A**) Growth curve of P19SCs treated with FLO. The results were expressed as the average cell numbers from three independent experiments. (**B**) Heat map of the DEGs enriched in the term “cell cycle G1/S phase transition”. Expression values of the six libraries are expressed as the normalized FPKM values. Red colors indicate upregulated DEGs. Blue colors indicate downregulated DEGs. The RNA-sequencing data used to assess the changes of cell cycle-related genes and BP in P19SCs post FLO treatment were downloaded from China National Center for Bioinformation under accession number CRA005030 (https://ngdc.cncb.ac.cn/gsa, accessed on 10 April 2023). (**C**) Apoptosis was, respectively, detected by DAPI staining or transmission electron microscopy. The red arrows and triangles indicate chromatin marginalization and condensation. Bar = 2 μm. ** *p* < 0.01, as compared with the control group. *ns*, not significant.

In a previous study, we observed that FLO suppresses the development of early chick embryos. The proliferation and differentiation of embryonic stem cells are intricately regulated processes that are critical for embryonic development. To understand how FLO affects stem cell proliferation during embryonic development, we treated P19SCs with FLO. As depicted in Figure 1A, the growth curve of FLO-treated P19SCs displayed a significant divergence from that of untreated cells, indicating an inhibitory effect of FLO on proliferation and self-renewal.

To further explore the alterations in cell cycle-related genes and BP in P19SCs following FLO treatment, we conducted high-throughput sequencing. The results revealed significant enrichment of the term “cell cycle G1/S phase transition” (adjusted *p* = 0.028) among the differentially expressed genes (DEGs), with a total of 39 DEGs involved (comprising 15 upregulated DEGs and 24 downregulated DEGs) (Figure 1B). Given that the precise transition from G1 phase of the cell cycle to S phase is pivotal for controlling stem cell proliferation, we postulated that FLO hinders P19SC proliferation by disrupting the G1/S phase transition.

Cell cycle arrest is one of the most prominent contributors to apoptosis. Indeed, we observed that a high dose of FLO induced the morphological characteristics of apoptosis, including chromatin marginalization and condensation (Figure 1C). Collectively, these findings suggest that FLO inhibits P19SC proliferation by perturbing the G1/S phase transition.

### 2.2. FLO Inhibits Cardiac Differentiation of P19SCs

Regarding the adverse effects of FLO on normal heart development in chicken embryos, the current literature on the impact of FLO on cardiac-specific differentiation is exceedingly limited. In this study, we employed P19SCs, a pluripotent embryonal carcinoma cell line capable of differentiating into three germ layers, as a model to investigate the cardiac toxicity of FLO.

**Figure 2:**
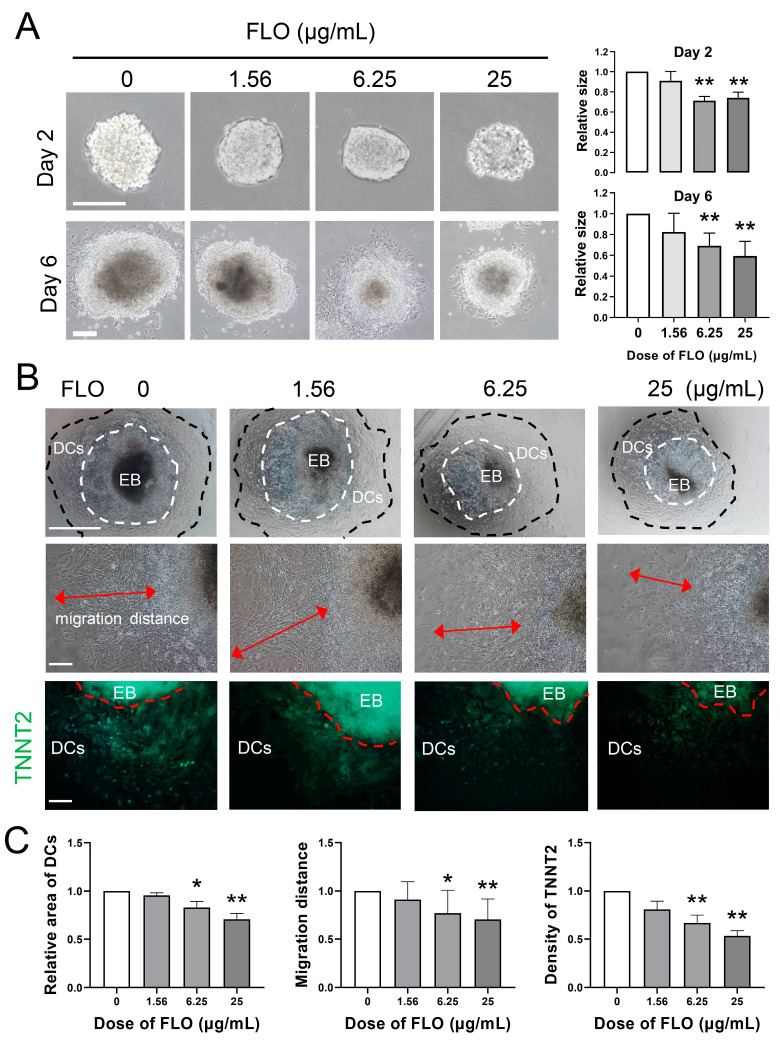
FLO inhibits cardiac differentiation of P19SCs. (**A**) The morphology and size of differentiated EBs (day 2 or day 6) post FLO treatment. Representative images are shown. Bar = 200 μm. (**B**) The morphology and differentiation degree of EBs on the 9th day of FLO-treated P19SCs and immunofluorescence staining of cardiomyocyte-specific marker protein TNNT2 (FITC labeled). EB, embryoid body; DCs, differentiated cells. The arrow indicates the extension range of differentiated cells. The white or red dashed line delineates the boundary between differentiated and undifferentiated cells. The black dashed line delineates the boundary of differentiated cells. Bar = 200 μm. (**C**) Bar graphs showed the area of DCs, migration distance, and density of TNNT2. * *p* < 0.05, ** *p* < 0.01, as compared with the control group.

The differentiation of P19SCs into cardiomyocytes was initiated by allowing the cells to aggregate in suspension culture in the presence of 1% DMSO. As illustrated in Figure 2A, incubation with FLO for two days significantly impeded the formation of aggregates, known as EBs. After four days of suspension culture, the EBs were transferred to tissue culture dishes for adherent culture to facilitate further growth. The reduced size of day 6 EBs further confirmed the inhibitory effect of FLO on P19SC differentiation (Figure 2A). After an additional three days of adherent culture (day 9), the differentiated cells (DCs) exhibited denser cytoplasm and migrated outward from the EBs. As depicted in Figure 2B, EBs exposed to FLO contained fewer DCs with shorter migration distances. To validate the differentiation of P19SCs into mature cardiomyocytes, we assessed the expression of TNNT2, a protein exclusive to cardiomyocytes, and observed that FLO decreased the proportion of cardiomyocytes among differentiated cells (Figure 2B,C). In summary, these findings suggest that FLO inhibits the cardiac differentiation of P19SCs.

### 2.3. Overview of Proteome Alterations Induced by FLO Treatment

P19SCs were treated with 10 μg/mL FLO for 48 h, and iTRAQ-based proteomic analysis was conducted to elucidate the mechanism underlying FLO-induced stem cell toxicity and evaluate the overall protein changes in P19SCs following FLO exposure. In summary, 593,965 spectra were obtained, corresponding to 53,595 unique peptides. Ultimately, 6002 proteins were identified with a false discovery rate of ≤0.01 (see Supplementary Table S1).

To quantitatively visualize the relationship between the six samples, a principal component analysis was performed based on the expression data of individual replicates. As depicted in Figure 1A, individual replicates from the two groups consistently formed two distinct clusters, underscoring the reliability of the data. Subsequently, DEPs were identified in the samples. The mean protein ratio in each group was used to calculate the protein expression ratio between the FLO-treated and untreated cells. A ratio with a fold change (FC) > 1.20 or <0.83 and *p* < 0.05 in the FLO group compared to the control group was considered a DEP.

**Figure 3:**
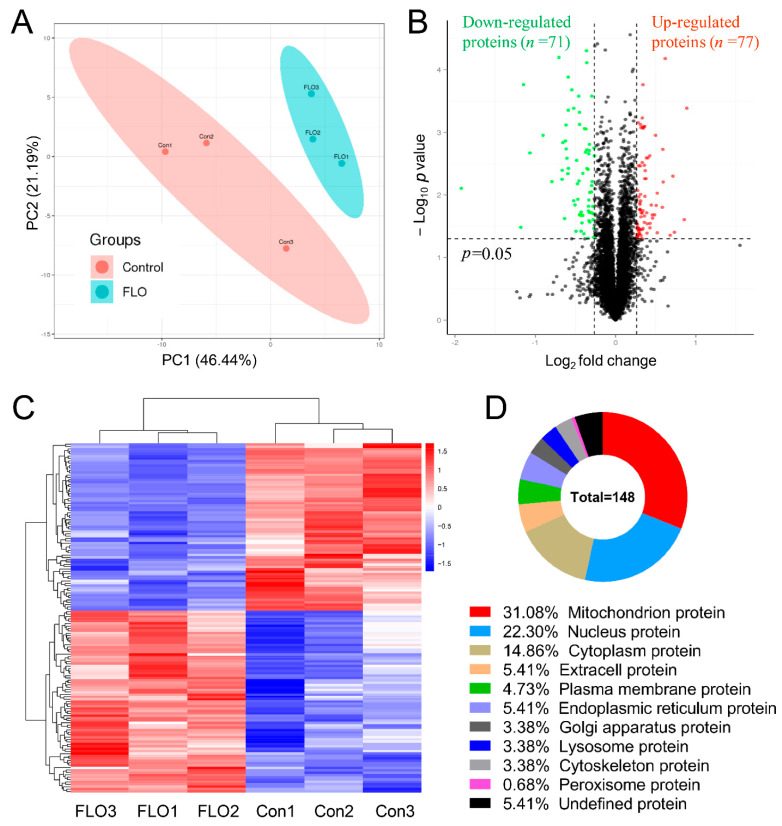
Proteomic alterations in P19SCs post FLO treatment. (**A**) Principal component analysis score chart of the six samples. (**B**) Volcano plot of DEPs. The red color indicates upregulated proteins (*p* < 0.05 and fold change > 1.2). Green color indicates downregulated proteins (*p* < 0.05 and fold change < 0.83). (**C**) Heatmap of the DEPs. The red color indicates upregulated DEPs. The blue color indicates downregulated DEPs. (**D**) The majority of DEPs were grouped into 10 subcellular components based on their subcellular localization.

Based on the LC-MS/MS data, 148 proteins were identified as DEPs and were hierarchically clustered into two groups, encompassing 77 upregulated and 71 downregulated proteins following FLO treatment (see Figure 3B,C, and Supplementary Table S2). Furthermore, the majority of these DEPs were categorized into ten subcellular components based on their subcellular localization (Figure 3D). Notably, 46 DEPs (31.08%) were identified as mitochondrial proteins, followed by nuclear proteins (*n* = 33, 22.30%), and cytoplasmic proteins (*n* = 22, 14.86%).

### 2.4. GO Functional Analysis of DEPs

**Figure 4:**
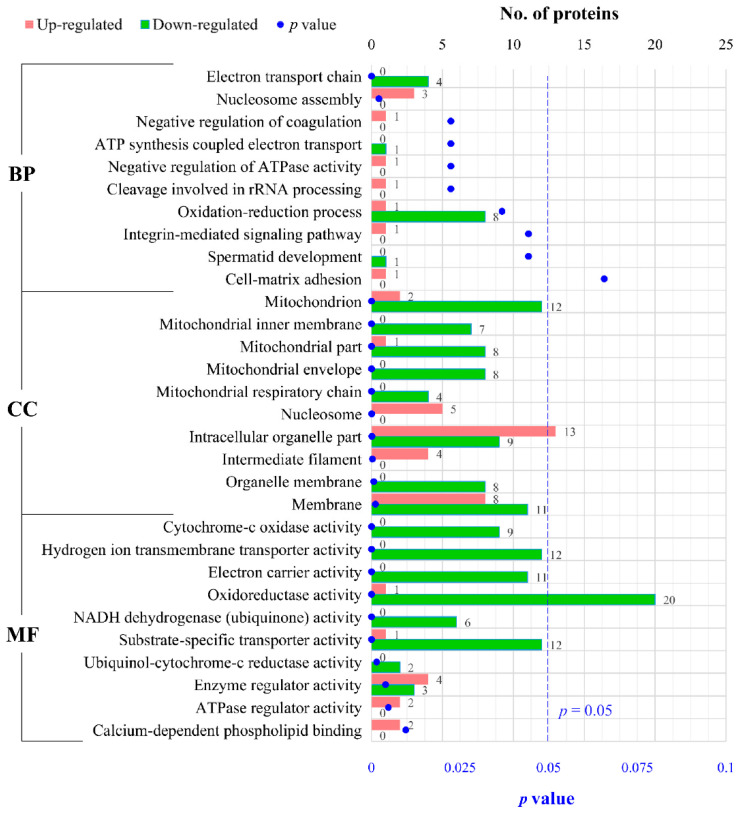
GO functional analysis of DEPs. The top 10 enriched GO terms of BP, CC, and MF were sorted according to p values. The number of upregulated (red) or downregulated (green) DEPs related to each term is shown.

The ClusterProfile tool was employed to analyze the dysregulated proteins, aiming to uncover alterations in BP, cell components (CC), and molecular functions (MF) in FLO-treated P19SCs, providing a comprehensive understanding of the 148 DEPs. A total of 58 BPs were enriched in this study, with 9 displaying statistical significance (*p* < 0.05). In Figure 4, the top 10 enriched terms sorted by *p*-value are presented. Notably, significantly enriched BP terms such as “electron transport chain”, “ATP synthesis coupled electron transport”, “negative regulation of ATPase activity”, and “oxidation-reduction process” predominantly revolved around oxidation–reduction reactions within mitochondria. This further underscores the importance of mitochondrial oxidative phosphorylation as a pivotal process that is affected by FLO.

In the context of CC ontology, 41 components were enriched, with 21 CC terms demonstrating statistical significance (*p* < 0.05). Figure 4 showcases the top 10 terms based on *p*-value, which primarily pertained to mitochondrial components, including “mitochondrion”, “mitochondrial inner membrane”, “mitochondrial part”, “mitochondrial envelope”, and “mitochondrial respiratory chain”.

As for the MF ontology, 56 MF terms were enriched, and 27 of them reached statistical significance (*p* < 0.05). It is evident that the altered protein functions were associated with “cytochrome-c oxidase activity”, “hydrogen ion transmembrane transporter activity”, “electron carrier activity”, “oxidoreductase activity”, “NADH dehydrogenase (ubiquinone) activity”, and others (Figure 4).

Collectively, the results from the GO functional analysis strongly indicate that FLO disrupts mitochondrial components and related BP, underscoring its role in cytotoxicity.

### 2.5. KEGG Pathway Analysis of DEPs

**Figure 5:**
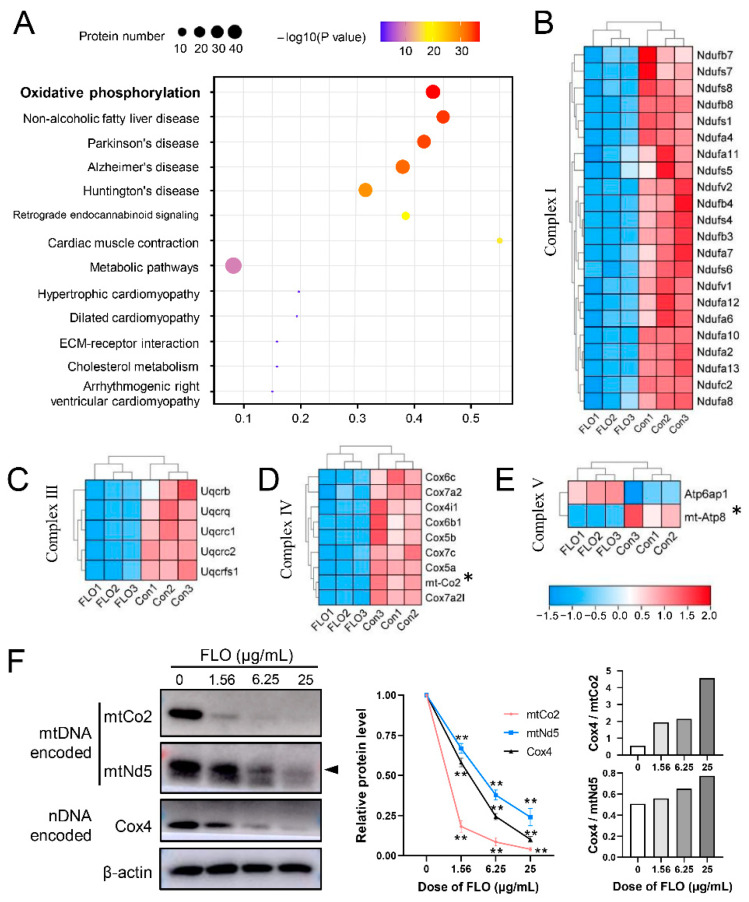
KEGG pathway enrichment analysis of DEPs and validation of proteomic results using Western blot. (**A**) Thirteen significantly enriched KEGG pathways of DEPs are shown. The size or color of the dots represent the number or significance of DEPs enriched in the GO term, respectively. A small *p* value corresponded to the great difference. (**B**–**E**) The relative levels of the identified DEPs of mitochondrial complex I, III, IV, and V in P19SCs post FLO treatment for 48 h. * indicates mtDNA-encoded protein. The red color indicates upregulated DEPs. The blue color indicates downregulated DEPs. (**F**) The expression levels of mtDNA-encoded proteins (mtCo2 and mtNd5) and ntDNA-encoded protein (Cox4) in FLO-treated P19SCs. Representative blots are shown and β-actin served as a loading control. Densitometry of the representative blots was performed for quantification and the ratios of Cox4 to mtCo2 or Cox4 to mtNd5 are presented to demonstrate mitonuclear protein imbalance. ** *p* < 0.01, as compared with the control group.

To gain insights into the specific biological events associated with dysregulated proteins, KEGG-based pathway analysis was conducted to gather information pertaining to protein functions in metabolic processes. A total of 70 enriched KEGG pathways were identified, with 13 pathways exhibiting significant enrichment (*p* < 0.05) (Figure 5A). Among these 13 significantly enriched pathways, five were enriched by more than 35 DEPs, including “Oxidative phosphorylation” (*p* = 2.95 × 10^−37^, 38 DEPs) and “Metabolic pathways” (*p* = 8.83 × 10^−10^, 43 DEPs).

Of particular interest is the “oxidative phosphorylation” pathway, which is an evolutionarily conserved process crucial for the proliferation and maintenance of stem cells and embryogenesis. This pathway encompasses 85 proteins, including 38 DEPs. Among these, one was upregulated, and the remaining 37 were downregulated (Supplementary Table S3). Specifically, 22 DEPs were subunits of mitochondrial complex I (NADH dehydrogenase; Figure 5B), 5 belonged to complex III (cytochrome c reductase; Figure 5C), 9 were associated with complex IV (cytochrome c oxidase; Figure 5D), and 2 were part of complex V (ATP synthase; Figure 5E). Notably, most of these DEPs were encoded by nuclear DNA (nDNA), with the exception of 2 DEPs (mt-Co2 and mt-Atp8), which are encoded by mitochondrial DNA (mtDNA).

In summary, these findings suggest for the first time that FLO inhibits the expression of both nDNA-encoded and mtDNA-encoded subunits of the electron transport chain, consequently suppressing oxidative phosphorylation.

### 2.6. Validation of Proteomic Results

To validate the findings obtained from the iTRAQ analysis, proteins encoded by nDNA, such as Cox4, or mtDNA, such as mtCo2, were selected for confirmation via Western blot analysis. As illustrated in Figure 5F, the protein levels of these mitochondrial subunits were reduced following FLO treatment, with a more pronounced decrease observed at higher FLO doses. These results are in agreement with the results of iTRAQ-based proteomic analyses.

Notably, the protein level of mtDNA-encoded mtNd5 also exhibited a reduction after FLO treatment, despite not being identified as DEP in the iTRAQ analysis (*p* = 0.263). The increased ratio of nDNA-encoded subunits to mtDNA-encoded subunits has recently been termed “mitonuclear protein imbalance”. Such an imbalance can influence stem cell functions, including survival, self-renewal, proliferation, and differentiation, in addition to its effects on respiration. Consequently, we calculated the ratios of Cox4/mtCo2 and Cox4/mtNd5 and found elevated ratios in FLO-treated cells (Figure 5F). These findings indicate that FLO induces a significant mitonuclear protein imbalance in P19SCs.

### 2.7. FLO Induced Mitochondrial Hyperfusion and Unfolded Protein Response

**Figure 6:**
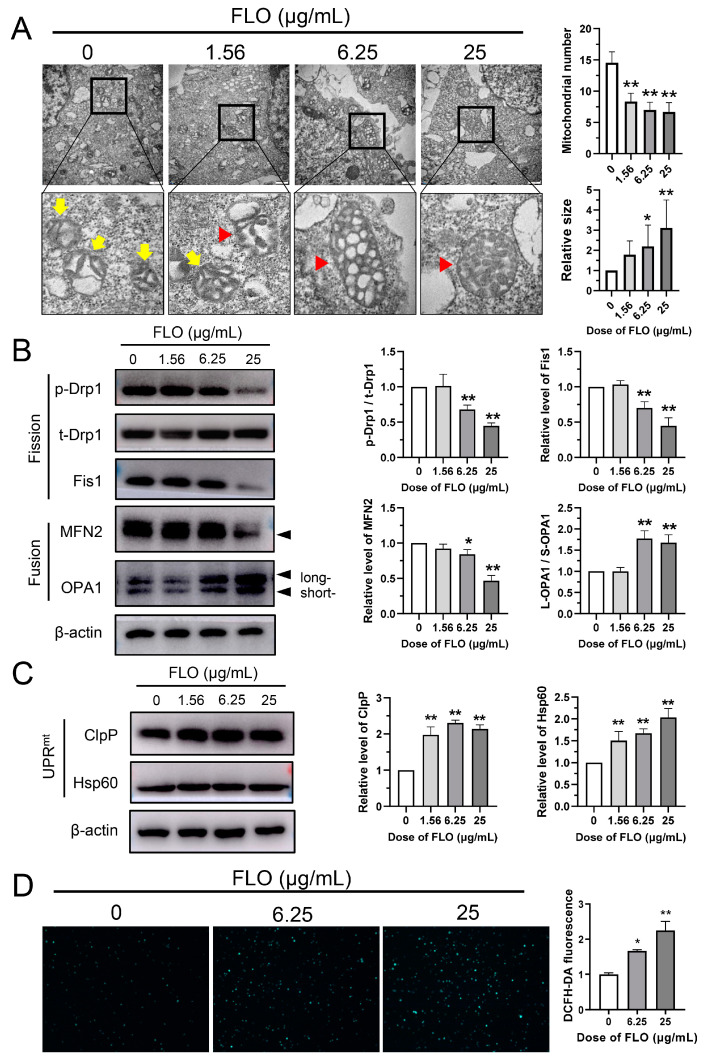
FLO-induced mitochondrial damage and abnormal mitochondrial dynamics in P19SCs. (**A**) Representative transmission electron images showing the mitochondrial ultrastructure changes in P19SCs treated with FLO for 48 h. The yellow arrows indicate mitochondria with disturbed cristae structure, while the red triangles indicate larger mitochondria. Quantification of the mitochondrial number (30,000×) and relative mitochondrial size (30,000×) are shown. Data are shown as mean ± SD. (**B**) The expression levels of mitochondrial fission factors (p-Drp1, t-Drp1, Fis1) and fusion factors (MFN2 and OPA1) post FLO treatment were detected by Western blot. Representative blots are shown and β-actin served as a loading control. Densitometry of the representative blots was performed for quantification. (**C**) The expression levels of UPRmt marker proteins ClpP and Hsp60 in P19SCs post FLO-treatment. Representative blots are shown. (**D**) ROS levels were detected with DCFH-DA fluorescent probe in FLO-treated P19SCs. *n* = 5. * *p* < 0.05, ** *p* < 0.01, as compared with the control group.

To gain further insight into how FLO-induced mitonuclear protein imbalance affects mitochondrial morphology, which is closely linked to energy status and cell viability, we conducted TEM observations of FLO-treated P19SCs. In the control group, mitochondria exhibited typical features of normal morphological characteristics in undifferentiated stem cells, appearing small and round (Figure 6A). However, in P19SCs treated with FLO, a series of pathological changes were evident, including increased elongation, larger mitochondrial size, and disrupted mitochondrial cristae structure. Furthermore, a significant dose-dependent reduction in mitochondrial numbers was observed in FLO-treated cells compared to that in the control group (Figure 6A). These findings indicate that FLO not only damages the mitochondrial ultrastructure but also results in fewer and larger mitochondria in P19SCs.

Mitochondria are dynamic organelles and their morphology is governed by a balance between fusion and fission processes. To investigate whether the altered mitochondrial morphology was accompanied by disruptions in mitochondrial dynamics, we examined four key components of the mitochondrial dynamics machinery in FLO-treated P19SCs. The results revealed that FLO dose-dependently inhibited the expression of the mitochondrial fission factors Drp1 and Fis1, while increasing the expression of the mitochondrial fusion factor OPA1. Notably, FLO also reduced the protein levels of MFN2, a dynamin-related GTPase essential for mitochondrial fusion (Figure 6B). These findings suggest that FLO disrupts mitochondrial dynamics, which could be a crucial factor contributing to the changes in mitochondrial morphology and quantity.

Moreover, we observed that FLO upregulated the expression of the mitochondrial chaperones ClpP and Hsp60 (Figure 6C). This indicates that FLO induces mitochondrial stress and activates the mitochondrial unfolded protein response, which maintains mitochondrial homeostasis and reduces the accumulation of misfolded proteins within the organelles. Furthermore, the treatment of FLO significantly increased reactive oxygen species (ROS) production in P19SCs (Figure 6D), which supports the notion that FLO damages mitochondrial function.

## 3. Discussion

FLO, an efficient and broad-spectrum antibacterial drug, has recently gained popularity in animal husbandry and aquaculture. However, it is also a drug with a high detection rate in eggs and poultry meat, making it a significant food safety concern [21,22]. Additionally, owing to its widespread use in livestock and aquaculture, the pollution and residues of FLO in aquaculture wastewater and natural waters pose potential public health risks [23,24,25]. In recent years, owing to the continuous detection of FLO residues in water environments and food, research on the toxicity of FLO has gained significant attention, including its hematopoietic toxicity, immunotoxicity, and embryonic toxicity [12,13,26,27]. Previous studies by our group found that FLO could delay the development of chicken embryos and impair mitochondrial function [13,15]. Therefore, it is crucial to elucidate the mechanism of embryonic toxicity induced by FLO for the rational use and development of new drugs. In this study, we observed that FLO inhibited the proliferation and differentiation of P19SCs and identified DEPs and mitochondria-related pathways involved in the FLO-induced cytotoxicity of P19SCs.

We employed the pluripotent cell line P19SCs, which is capable of differentiating into three germ layers, as a model to assess the impact of FLO on the proliferation and differentiation of ESCs. Our findings revealed that FLO inhibited the proliferation and self-renewal of P19SCs (Figure 1A). Cell cycle regulation plays a critical role in cell proliferation, growth, and repair and involves a complex network of proteins, enzymes, cytokines, and signaling pathways [28]. Apoptosis and proliferation are interconnected through cell cycle regulation, and apoptosis induction simultaneously affects both cell proliferation and cell death [16]. Our high-throughput sequencing results indicated that the term “G1/S phase transition of the cell cycle” was significantly enriched by 39 differentially expressed genes (Figure 1B). Furthermore, higher concentrations of FLO induced the apoptosis of P19SCs (Figure 1C). These results suggest that FLO inhibits P19SC proliferation by interfering with G1/S phase transition. Our previous research has reported that FLO affects the proliferation and pluripotency of P19SCs [29]. The current study further reinforces the role of the cell cycle transition in cell proliferation. Notably, in a previous study, we observed that FLO had a detrimental effect on the normal development of the heart in chick embryos, as indicated by a reduced transverse cardiac diameter [13]. However, the mechanisms underlying FLO-induced cardiac impairment remain unclear. In the present study, we found that FLO inhibited the directed differentiation of P19SCs into cardiomyocytes (Figure 2A,C), which could explain the impaired heart development observed in chicken embryos exposed to FLO.

To elucidate the mechanism underlying FLO-induced stem cell toxicity and to comprehensively assess changes in protein expression in FLO-treated P19SCs, we conducted iTRAQ-based quantitative proteome analysis. The results revealed that 31.08% of the DEPs (46 out of 148) were classified as mitochondrial proteins (Figure 3D), followed by nuclear proteins (*n* = 33, 22.30%), suggesting that the primary organelles affected by FLO are mitochondria. This observation is likely related to FLO’s binding to the large subunit of mitoribosomes and its inhibition of peptidyl transferase in the mitochondria. Furthermore, our analysis indicated that DEPs were significantly enriched in terms of mitochondrial redox reactions, including “the electron transport chain”, “ATP synthesis-coupled electron transport”, and “negative regulation of ATPase activity” (Figure 4). KEGG pathway analysis highlighted that the most enriched pathway for these DEPs was the “oxidative phosphorylation pathway” (Figure 5A). These findings further support the notion that mitochondrial oxidative phosphorylation, an evolutionarily conserved pathway involved in stem cell proliferation and embryogenesis [30], may play a critical role in FLO’s impact. Mitochondrial dysfunction triggered by chemical agents can inhibit pluripotency, differentiation potential, and embryonic development potential in pluripotent stem cells [7,16,31]. In summary, these results underscore the significance of mitochondrial damage and dysfunction in FLO-induced disturbances of stem cell differentiation and embryonic development.

Eukaryotic mitochondria contain approximately 1500 proteins, of which mtDNA encodes 13 protein subunits that are involved in the oxidative phosphorylation pathway. These subunits are essential components of the mitochondrial respiratory chain complexes (I, III, and IV) [32]. Most other proteins are encoded by nDNA and are subsequently transported to the mitochondria. Typically, peptides and proteins encoded by nDNA and mtDNA undergo synchronous and complementary post-translational modifications, ultimately forming mitochondrial respiratory chain complexes. In this process, an increase in the ratio of nDNA-encoded proteins to mtDNA-encoded proteins leads to a condition known as mitonuclear protein imbalance. This imbalance can result in the incorrect assembly of mitochondrial respiratory chain complexes [33], leading to protein toxicity and metabolic stress within the mitochondria. These stresses can affect the survival, self-renewal, proliferation, and differentiation of stem cells [31,34]. Numerous studies have reported that antibiotics, such as tetracyclines, aminoglycosides, macrolides, and chloramphenicol, can inhibit mitochondrial protein synthesis, thereby damaging the mitochondrial respiratory chain [35,36].

In this study, we confirmed that FLO binding to the mitochondrial large subunit significantly reduced the protein levels encoded by mtDNA in P19SCs, resulting in a mitonuclear protein imbalance (Figure 5F). Transmission electron microscopy results demonstrated that FLO not only damaged the ultrastructure of mitochondria but also increased their volume while decreasing their number in P19SCs (Figure 6A). Consequently, it is plausible to speculate that mitochondrial damage is a primary cause of FLO-induced embryonic toxicity.

Mitochondria maintain their normal quantity, shape, and function through continuous fusion and division, a process known as mitochondrial dynamics [37]. The balance of mitochondrial dynamics relies on the coordinated regulation of mitochondrial fusion and fission proteins, which play crucial roles in tissue formation and embryonic development [38]. Disturbing the balance of mitochondrial dynamics by inhibiting fission factor Drp1 can disrupt postnatal development [39] and suppress the expression of key myogenic regulatory factors during myogenic differentiation [40]. Besides mitochondrial fission, mitochondrial fusion is also essential for cellular differentiation processes. Deletion of the fusion factor MFN2 results in the defective cardiac differentiation of ESCs, impaired mouse heart development, and neuronal differentiation [41,42].

In this study, FLO inhibited the expression of the mitochondrial fission factor Drp1 and mitochondrial fusion factor MFN2, indicating that FLO disrupted mitochondrial dynamics in P19SCs.Organisms have evolved a mitochondrial unfolded protein response (UPR^mt^) to induce the transcription of mitochondrial chaperones and proteases when faced with mitochondrial stress, ultimately restoring mitochondrial protein homeostasis [43]. In the current study, treatment with FLO induced mitonuclear protein imbalance and abnormal mitochondrial dynamics while increasing the expression of ClpP and Hsp60 (Figure 6C), both of which are markers of UPR^mt^. These findings further corroborate the notion that FLO-induced protein disequilibrium and mitochondrial dysfunction play a role in the inhibition of P19SC proliferation and directional differentiation into cardiomyocytes.

## 4. Materials and Methods

### 4.1. Chemicals

Minimum essential medium α (α-MEM) was procured from Procell Life Science & Technology Co., Ltd. (Wuhan, China). Fetal calf serum and bovine calf serum were sourced from Biological Industries (Kibbutz Beit Haemek, Israel). The FLO was obtained from Aladdin (Shanghai, China). Dimethyl sulfoxide (DMSO) was purchased from Sigma (St. Louis, MO, USA).

### 4.2. Cell Culture and Drug Treatment

P19SCs (catalog number CL-0179) were procured from Procell Life Science & Technology Co., Ltd. (Wuhan, China) and cultured in α-MEM medium, which included 7.5% calf serum, 2.5% fetal calf serum, 1 IU/mL penicillin, and 1 μg/mL streptomycin. The cells were maintained at 37 °C in a humidified incubator with 5% CO_2_. Cells were subjected to different treatments to reach a specific confluence threshold. The FLO concentrations employed in this study (ranging from 1.56 to 25 μg/mL) mirror those encountered in developing embryos or cells [29].

### 4.3. Differentiation of P19SCs

To induce cardiac mesodermal differentiation, P19 cells were suspended in a differentiation medium (growth medium containing 1% DMSO), and FLO was introduced into the differentiation medium. The cell suspension was then placed on the inverted inner surface of the culture dish cover and incubated for two days (day 2). Subsequently, the cell suspension was transferred to 6 cm cell culture dishes coated with gelatin, and fresh differentiation medium containing FLO was added. After 4 days (day 6), the resulting embryoid bodies (EBs) were transferred to 6-well culture plates filled with fresh differentiation medium containing FLO for adherent culture and further cultured for an additional 3 days (day 9). Morphological changes in the P19SCs were observed and photographed using an inverted microscope.

### 4.4. Cell Growth Curve and Cell Cycle-Related Genes Analysis

P19SCs were trypsinized, evenly distributed into each well of a 24-well plate, and incubated overnight. Subsequently, the cells were exposed to a concentration of 6.25 μg/mL FLO. At intervals of 12 h, the culture medium was aspirated, and adherent cells were detached using trypsin and then quantified using an Automated cell counter (IC1000, Ruiyu Biotech Co. Ltd., Shanghai, China). The cell counts from three wells at each time point were averaged within each group, and these data were used to generate the growth curves.

To evaluate changes in cell cycle-related genes and BP in P19SCs following FLO treatment, RNA sequencing data were retrieved from the China National Center for Bioinformation, accessible under accession number CRA005030 (https://ngdc.cncb.ac.cn/gsa, accessed on 10 April 2023). The RNA-sequencing dataset was analyzed using the methodologies described in our previously published study [29].

### 4.5. Apoptosis Assay

The apoptosis rate in the treated cells was assessed by DAPI staining. Briefly, cells were seeded on sterile cover glasses in 12-well culture plates prior to treatment. After 48 h of FLO exposure, cells were washed with PBS. Subsequently, DAPI solution (2 μg/mL) was applied and incubated at room temperature for 5 min, followed by a PBS rinse. The coverslips were mounted onto slides using an anti-fluorescence inhibitor. Images were acquired using a fluorescence microscope (Nikon, TI-FL, Tokyo, Japan).

### 4.6. Transmission Electron Microscope

The P19SCs were harvested after FLO treatment. They were initially fixed with 2.5% glutaraldehyde at low temperature, followed by treatment with 1% osmium tetroxide until the cells were fixed. Subsequently, they were dehydrated using a graded alcohol series. Subsequently, the cells were embedded in araldite resin, sectioned, stained with uranyl acetate and lead citrate, and examined using a transmission electron microscope (TEM, H-7500, Hitachi, Tokyo, Japan).

### 4.7. Immunofluorescence Staining

After 9 days of P19SC differentiation, the medium was aspirated and the cells were fixed for 15 min in 4% (*w*/*v*) paraformaldehyde at room temperature. Subsequently, the cells were washed three times with PBS, followed by permeabilization with 0.1% Triton X-100 in cold PBS for 15 min. After another three PBS washes, the cells were blocked with 1% BSA for 1 h at room temperature. Following this, the cells were incubated with primary antibodies against TNNT2 (Table S4) at 4 °C overnight.

The cells were again washed three times with cold PBS and then incubated with a Fluorescein Isothiocyanate secondary antibody diluted in 5% BSA for 1 h at room temperature. The cell nuclei were stained with 2 μg/mL DAPI (Solarbio, Beijing, China) for 5 min in the dark, followed by three washes with cold PBS. Subsequently, the coverslip was mounted on a slide using an anti-fluorescence inhibitor. The cells were imaged using a laser confocal microscope (LSM 800, Zeiss, Jena, Germany) and the image data were analyzed using ImageJ software (V 1.8.0, National Institutes of Health, New York, MD, USA).

### 4.8. Western Blot Analysis

After FLO treatment, cells were washed with cold PBS and lysed using RIPA lysis buffer (Solarbio, Beijing, China). The whole cell lysates were prepared for Western blot analysis. Protein concentrations were determined using the BCA protein concentration detection kit (Beyotime, Beijing, China). Equal amounts of protein (30 μg) were separated using SDS-PAGE and transferred to a polyvinylidene fluoride (PVDF) membrane. The membrane was blocked with a PBST solution containing 5% skim milk for 2 h.

Next, the membranes were incubated with the primary antibodies at 4 °C overnight. They were then washed six times for 5 min each with PBST and subsequently incubated with an IgG horseradish peroxidase-labeled secondary antibody (ZSGB-BIO, Beijing, China). After another six washes of 5 min each with PBST, the membranes were visualized using super-sensitivity chemiluminescence reagents (Willget Biotech, Shanghai, China).

### 4.9. iTRAQ Quantification and Bioinformatics Analysis

Total proteins were extracted by lysing cells in lysis buffer and subsequently alkylated with iodoacetamide. Protein quality was assessed. The digested peptides were desalted, lyophilized, and labeled with the iTRAQ labeling reagent. Lyophilized powder was dissolved and fractionated using a C18 column. All the fractions were dried under vacuum and reconstituted in 0.1% formic acid in water. To construct the transition library, shotgun proteomics analyses were performed using an EASY-nLC™ 1200 UHPLC system (Thermo Fisher, Waltham, MA, USA) coupled with a Q Exactive™ HF-X mass spectrometer (Thermo Fisher) operating in data-dependent acquisition mode (Novogene, Beijing, China).

The resulting spectra from each run were individually searched against a Mus Musculus database using Proteome Discoverer 2.2 (PD 2.2, Thermo). Proteins that exhibited significant differences in quantitation between the experimental and control groups (*p* < 0.05 and fold change > 1.20 or <0.83) were classified as DEPs. Gene Ontology (GO) functional analysis was performed using the InterProScan program (InterPro 97.0, EMBL’s European Bioinformatics Institute, Hinxton, Cambridgeshire, UK) (http://www.ebi.ac.uk/interpro/, accessed on 9 November 2023) against the non-redundant protein database [44], and the Kyoto Encyclopedia of Genes and Genomes (KEGG) database was used to analyze enriched pathways. The sequencing data were deposited in the integrated proteome resource iProX, National Center for Protein Sciences (Beijing, China), under the project ID IPX0003535000 (https://www.iprox.cn/, accessed on 24 May 2023).

### 4.10. ROS Assay

ROS levels of cells were measured by using a fluorescent probe, 2′,7′-dichlorodihydrofluorescein (DCFH) (Beyotime), which promptly converts into the highly fluorescent 2′,7′-dichlorofluorescein (DCF) in the presence of intracellular ROS. The monitoring of fluorescence was carried out through a fluorescence microscope (Nikon, Tokyo, Japan) at 488 nm. The quantity of ROS was calculated as the relative fluorescence intensity of DCF per cell in the scan area.

### 4.11. Statistical Analysis

The data were analyzed using one-way ANOVA followed by the LSD multiple comparison test, conducted using SPSS software (version 20.0; SPSS, Chicago, IL, USA). Each experiment was independently conducted at least three times, and the data are presented as mean ± SD. Statistical significance was set at *p* < 0.05.

## 5. Conclusions

In this study, we provided evidence of decreased proliferation viability and suppressed cardiac differentiation in FLO-treated P19SCs. These findings contribute to the understanding of how FLO induces abnormal cardiac development and embryonic toxicity. Furthermore, alterations in the abundance of several proteins primarily related to mitochondrial components were observed. These proteins were enriched in BP related to oxidation–reduction reactions in the mitochondria, suggesting that mitochondrial oxidative phosphorylation may be a crucial process affected by FLO. Additionally, our results highlight the presence of a mitonuclear protein imbalance, damage to the mitochondrial ultrastructure, the presence of fewer and larger mitochondria, and abnormal mitochondrial dynamics in FLO-treated P19SCs. Collectively, these findings indicate that mitochondria are a key target of FLO toxicity.

In summary, this study provides a comprehensive characterization of the mitochondrial responses to FLO in P19SCs, enhancing our understanding of the molecular mechanisms underlying FLO-induced embryonic toxicity. Further investigations into the roles of the identified proteins and studies in animal models are needed to fully elucidate the contribution of these mitochondrial responses to FLO-induced embryonic toxicity.

## Data Availability

The data presented in this study are available in the Supplementary Material. The sequencing data were deposited in the integrated proteome resource iProX, National Center for Protein Sciences (Beijing, China), under the project ID IPX0003535000 (https://www.iprox.cn/, accessed on 24 May 2023).

## Supplementary Materials

The following supporting information can be downloaded at: https://www.mdpi.com/article/10.3390/toxics11120992/s1, Table S1: the identified proteins; Table S2: differentially expressed proteins; Table S3: DEPs enriched in oxidative phosphorylation pathway; Table S4: antibodies used in this study.

## References

[1] Gaudias J. (2020). Antibiotic prophylaxis in orthopedics-traumatology. Orthop. Traumatol. Surg. Res..

[2] Guidi L.R., Tette P.A., Fernandes C., Silva L.H., Gloria M.B.A. (2017). Advances on the chromatographic determination of amphenicols in food. Talanta.

[3] Shi X., Xia Y., Wei W., Ni B.-J. (2022). Accelerated spread of antibiotic resistance genes (ARGs) induced by non-antibiotic conditions: Roles and mechanisms. Water Res..

[4] Apreja M., Sharma A., Balda S., Kataria K., Capalash N., Sharma P. (2022). Antibiotic residues in environment: Antimicrobial resistance development, ecological risks, and bioremediation. Environ. Sci. Pollut. Res. Int..

[5] Manyi-Loh C., Mamphweli S., Meyer E., Okoh A. (2018). Antibiotic Use in Agriculture and Its Consequential Resistance in Environmental Sources: Potential Public Health Implications. Molecules.

[6] Müller M.M., Nedielkov R., Arndt K.M. (2022). Strategies for Enzymatic Inactivation of the Veterinary Antibiotic Florfenicol. Antibiotics.

[7] Liu D., Liu N.Y., Chen L.T., Shao Y., Shi X.M., Zhu D.Y. (2020). Perfluorooctane sulfonate induced toxicity in embryonic stem cell-derived cardiomyocytes via inhibiting autophagy-lysosome pathway. Toxicol. In Vitro.

[8] Wiest D.B., Cochran J.B., Tecklenburg F.W. (2012). Chloramphenicol Toxicity Revisited: A 12-Year-Old Patient With a Brain Abscess. J. Pediatr. Pharmacol. Ther..

[9] Wang X., Liu W., Liu Y., Jiao Y., Rong C., Liu Q., Shi W. (2022). Florfenicol induced renal inflammatory response and apoptosis via cell adhesion molecules signaling pathway. Poult. Sci..

[10] Wang X., Liu W., Zhang D., Jiao Y., Zhao Q., Liu Y., Shi W., Bao Y. (2023). Salvia miltiorrhiza polysaccharides alleviate florfenicol-induced inflammation and oxidative stress in chick livers by regulating phagosome signaling pathway. Ecotoxicol. Environ. Saf..

[11] Zhang L., Qiu J., Li Y., He L., Mao M., Wang T., Pan Y., Li Z., Mu X., Qian Y. (2023). Maternal transfer of florfenicol impacts development and disrupts metabolic pathways in F1 offspring zebrafish by destroying mitochondria. Ecotoxicol. Environ. Saf..

[12] Al-Shahrani S., Naidoo V. (2015). Florfenicol induces early embryonic death in eggs collected from treated hens. BMC Vet.-Res..

[13] Hu D., Meng F., Cui Y., Yin M., Ning H., Yin Z., Chen L., Ge Y., Liu S. (2020). Growth and cardiovascular development are repressed by florfenicol exposure in early chicken embryos. Poult. Sci..

[14] Roger A.J., Muñoz-Gómez S.A., Kamikawa R. (2017). The Origin and Diversification of Mitochondria. Curr. Biol..

[15] Hu D., Cao S., Zhang G., Xiao Y., Liu S., Shang Y. (2017). Florfenicol-induced Mitochondrial Dysfunction Suppresses Cell Proliferation and Autophagy in Fibroblasts. Sci. Rep..

[16] Kim Y.S., Yoon J.W., Kim D., Choi S., Kim H.K., Youm J.B., Han J., Heo S.C., Hyun S.-A., Seo J.-W. (2022). Tomatidine-stimulated maturation of human embryonic stem cell-derived cardiomyocytes for modeling mitochondrial dysfunction. Exp. Mol. Med..

[17] Martins W.K., Santos N.F., Rocha C.d.S., Bacellar I.O.L., Tsubone T.M., Viotto A.C., Matsukuma A.Y., Abrantes A.B.d.P., Siani P., Dias L.G. (2019). Parallel damage in mitochondria and lysosomes is an efficient way to photoinduce cell death. Autophagy.

[18] Lee S., Jeong Y., Roe J.-S., Huh H., Paik S.H., Song J. (2021). Mitochondrial dysfunction induced by callyspongiolide promotes autophagy-dependent cell death. BMB Rep..

[19] Morii A., Katayama S., Inazu T. (2020). Establishment of a Simple Method for Inducing Neuronal Differentiation of P19 EC Cells without Embryoid Body Formation and Analysis of the Role of Histone Deacetylase 8 Activity in This Differentiation. Biol. Pharm. Bull..

[20] Kanungo J. (2017). Retinoic Acid Signaling in P19 Stem Cell Differentiation. Anti-Cancer Agents Med. Chem..

[21] Liu F., Ma M., Li L., Wang M., Qin Y., Xu F. (2022). Investigation and health risk assessment of veterinary drug residues in chickens and eggs sold in Ningxia from 2016 to 2020. Wei Sheng Yan Jiu = J. Hyg. Res..

[22] Zhang H., Rong X., Wang C., Liu Y., Lu L., Li Y., Zhao C., Zhou J. (2020). VBP1 modulates Wnt/β-catenin signaling by mediating the stability of the transcription factors TCF/LEFs. J. Biol. Chem..

[23] Guo F., Wang Y., Peng J., Huang H., Tu X., Zhao H., Zhan N., Rao Z., Zhao G., Yang H. (2022). Occurrence, Distribution, and Risk Assessment of Antibiotics in the Aquatic Environment of the Karst Plateau Wetland of Yangtze River Basin, Southwestern China. Int. J. Environ. Res. Public Health.

[24] Zhang Y., Zhang X., Guo R., Zhang Q., Cao X., Suranjana M., Liu Y. (2020). Effects of florfenicol on growth, photosynthesis and antioxidant system of the non-target organism Isochrysis galbana. Comp. Biochem. Physiol. Part C Toxicol. Pharmacol..

[25] Zhang Y., Guo P., Wang M., Wu Y., Sun Y., Su H., Deng J. (2021). Mixture toxicity effects of chloramphenicol, thiamphenicol, florfenicol in Daphnia magna under different temperatures. Ecotoxicology.

[26] Liu W., Wang X., Liu Y., Fang S., Wu Z., Han C., Shi W., Bao Y. (2022). Effects of early florfenicol exposure on glutathione signaling pathway and PPAR signaling pathway in chick liver. Ecotoxicol. Environ. Saf..

[27] Mu Y., Lan M., Li Y., Zhang Z., Guan Y. (2023). Effects of florfenicol on the antioxidant and immune systems of Chinese soft-shelled turtle (Pelodiscus sinensis). Fish Shellfish. Immunol..

[28] Sun Y., Liu Y., Ma X., Hu H. (2021). The Influence of Cell Cycle Regulation on Chemotherapy. Int. J. Mol. Sci..

[29] Hu D., Zhang B., Suo Y., Li Z., Wan Z., Zhao W., Chen L., Yin Z., Ning H., Ge Y. (2022). Molecular Mechanisms Underlying the Inhibition of Proliferation and Differentiation by Florfenicol in P19 Stem Cells: Transcriptome Analysis. Front. Pharmacol..

[30] Paccola C., Souza G., Freitas I., Souza J., Martins L., Vendramini V., Miraglia S. (2021). Does maternal exposure to nicotine affect the oocyte quality and reproductive capacity in adult offspring?. Toxicol. Appl. Pharmacol..

[31] Khacho M., Clark A., Svoboda D.S., MacLaurin J.G., Lagace D.C., Park D.S., Slack R.S. (2017). Mitochondrial dysfunction underlies cognitive defects as a result of neural stem cell depletion and impaired neurogenesis. Hum. Mol. Genet..

[32] Zhao L., Sumberaz P. (2020). Mitochondrial DNA Damage: Prevalence, Biological Consequence, and Emerging Pathways. Chem. Res. Toxicol..

[33] Houtkooper R.H., Mouchiroud L., Ryu D., Moullan N., Katsyuba E., Knott G., Williams R.W., Auwerx J. (2013). Mitonuclear protein imbalance as a conserved longevity mechanism. Nature.

[34] Tournaire G., Loopmans S., Stegen S., Rinaldi G., Eelen G., Torrekens S., Moermans K., Carmeliet P., Ghesquière B., Thienpont B. (2022). Skeletal progenitors preserve proliferation and self-renewal upon inhibition of mitochondrial respiration by rerouting the TCA cycle. Cell Rep..

[35] Mottis A., Li T.Y., El Alam G., Rapin A., Katsyuba E., Liaskos D., D’amico D., Harris N.L., Grier M.C., Mouchiroud L. (2022). Tetracycline-induced mitohormesis mediates disease tolerance against influenza. J. Clin. Investig..

[36] Zhang Y., Fang Q., Wang H., Qi J., Sun S., Liao M., Wu Y., Hu Y., Jiang P., Cheng C. (2023). Increased mitophagy protects cochlear hair cells from aminoglycoside-induced damage. Autophagy.

[37] Adebayo M., Singh S., Singh A.P., Dasgupta S. (2021). Mitochondrial fusion and fission: The fine-tune balance for cellular homeostasis. FASEB J..

[38] Yapa N.M., Lisnyak V., Reljic B., Ryan M.T. (2021). Mitochondrial dynamics in health and disease. FEBS Lett..

[39] Wakabayashi J., Zhang Z., Wakabayashi N., Tamura Y., Fukaya M., Kensler T.W., Iijima M., Sesaki H. (2009). The dynamin-related GTPase Drp1 is required for embryonic and brain development in mice. J. Cell Biol..

[40] De Palma C., Falcone S., Pisoni S., Cipolat S., Panzeri C., Pambianco S., Pisconti A., Allevi R., Bassi M.T., Cossu G. (2010). Nitric oxide inhibition of Drp1-mediated mitochondrial fission is critical for myogenic differentiation. Cell Death Differ..

[41] Kasahara A., Cipolat S., Chen Y., Dorn G.W., Scorrano L. (2013). Mitochondrial Fusion Directs Cardiomyocyte Differentiation via Calcineurin and Notch Signaling. Science.

[42] Fang D., Yan S., Yu Q., Chen D., Yan S.S. (2016). Mfn2 is Required for Mitochondrial Development and Synapse Formation in Human Induced Pluripotent Stem Cells/hiPSC Derived Cortical Neurons. Sci. Rep..

[43] Wang S., Gao K., Liu Y. (2018). UPR mt coordinates immunity to maintain mitochondrial homeostasis and animal fitness. Mitochondrion.

[44] Jones P., Binns D., Chang H.-Y., Fraser M., Li W., McAnulla C., McWilliam H., Maslen J., Mitchell A., Nuka G. (2014). InterProScan 5: Genome-scale protein function classification. Bioinformatics.

